# Classification of Heart Failure Using Machine Learning: A Comparative Study

**DOI:** 10.3390/life15030496

**Published:** 2025-03-19

**Authors:** Bryan Chulde-Fernández, Denisse Enríquez-Ortega, Cesar Guevara, Paulo Navas, Andrés Tirado-Espín, Paulina Vizcaíno-Imacaña, Fernando Villalba-Meneses, Carolina Cadena-Morejon, Diego Almeida-Galarraga, Patricia Acosta-Vargas

**Affiliations:** 1 School of Biological Sciences and Engineering, Yachay Tech University, Hacienda San José s/n, San Miguel de Urcuquí 100119, Ecuador; bryan.chulde@yachaytech.edu.ec (B.C.-F.); denisse.enriquez@yachaytech.edu.ec (D.E.-O.); pnavas@yachaytech.edu.ec (P.N.); gvillalba@yachaytech.edu.ec (F.V.-M.); dalmeida@yachaytech.edu.ec (D.A.-G.); 2 Quantitative Methods Department, CUNEF University, 28040 Madrid, Spain; cesar.guevara@cunef.edu; 3 School of Mathematical and Computational Sciences, Universidad Yachay Tech, San Miguel de Urcuquí 100119, Ecuador; ctirado@yachaytech.edu.ec (A.T.-E.); ccadena@yachaytech.edu.ec (C.C.-M.); 4 Faculty of Technical Sciences, School of Computer Science, UIDE-International University of Ecuador, Quito 170501, Ecuador; pvizcaino@uide.edu.ec; 5 Intelligent and Interactive Systems Laboratory, Universidad de Las Américas, Quito 170125, Ecuador

**Keywords:** heart failure, machine learning, classification, feature extraction, diagnosis

## Abstract

Several machine learning classification algorithms were evaluated using a dataset focused on heart failure. Results obtained from logistic regression, random forest, decision tree, K-nearest neighbors, and multilayer perceptron (MLP) were compared to obtain the best model. The random forest method obtained specificity = 0.93, AUC = 0.97, and Matthews correlation coefficient (MCC) = 0.83. The accuracy was high; therefore, it was considered the best model. On the other hand, K-nearest neighbors and MLP (multi-layer perceptron) showed lower accuracy rates. These results confirm the effectiveness of the random forest method in identifying heart failure cases. This study underlines that the number of features, feature selection and quality, model type, and hyperparameter fit are also critical in these studies, as well as the importance of using machine learning techniques.

## 1. Introduction

Currently, cardiovascular diseases stand as one of the leading causes of global morbidity and mortality [1,2]. Detecting and diagnosing these conditions in a timely manner has become a critical challenge in the field of healthcare [3]. Cardiovascular diseases, also known as heart diseases, encompass a diverse group of conditions that impact the heart and blood vessels. These conditions can range from diseases affecting the coronary arteries (the arteries that supply blood to the heart) to disorders affecting heart valves, cardiac muscles, and the electrical conduction system of the heart [4,5].

Heart failure (HF) occurs when the heart cannot pump blood efficiently, affecting the delivery of oxygen and nutrients to tissues. It can be caused by coronary artery disease, hypertension, and damage to the heart muscle, presenting with extreme fatigue, shortness of breath, and edema [6,7,8]. Diagnosis is challenging due to the variety of clinical presentations and the similarity to other conditions, requiring imaging tests and biomarker monitoring for accurate diagnosis and treatment [9,10].

The significance of classifying individuals with heart failure from those without it lies in several crucial aspects [11,12]. First and foremost, the early detection of cardiovascular diseases enables timely medical intervention, which can be pivotal in improving patients’ prognosis. Early treatment can help prevent severe complications such as heart attacks or heart failure and reduce the disease burden associated with these conditions [13,14,15].

Furthermore, accurate identification of individuals at risk of heart failure is essential for the implementation of prevention and control strategies [16,17]. These strategies may include lifestyle changes, such as a healthy diet and regular exercise, as well as the management of risk factors, including high blood pressure and elevated cholesterol levels. Precise classification allows resources and efforts to be directed toward those who need them the most [18,19,20].

In this context, the use of machine learning (ML) algorithms has emerged as powerful tools to address this challenge [21,22]. This article explores the use of a dataset from the Kaggle platform named “Heart Failure”, consisting of approximately 10 crucial features for the classification of cardiovascular diseases [23]. These features provide key information on the cardiovascular health of a patient and serve as fundamental variables in the application of supervised learning and machine-learning techniques for the accurate classification of heart diseases [24,25].

This paper’s innovation resides in its implementation of machine learning algorithms for the prediction of heart failure, combining advanced preprocessing techniques, such as the elimination of outliers, and the optimization of hyperparameters to maximize the performance of the models. Its structured design ensures adaptability to different databases of other diseases, making it a valuable educational tool. The selection of a robust dataset and the integration of metrics such as specificity, AUC, and Brier score reinforce its clinical applicability.

By obtaining robust metrics such as specificity, AUC, and Brier score, the potential role of machine learning in early detection and clinical decision making is reinforced. More than just predicting heart failure with certain accuracy, the model can be integrated into the Clinical Decision Support System (CDSS), helping to identify high-risk patients in real time, to prioritize critical cases, and to optimize resources, enhancing hospital efficiency and clinical results.

## 2. Previous Works

In this section, a comprehensive literature review is conducted, focusing on cases related to the subject at hand. Various studies and methodologies are examined; the different machine learning techniques employed in this context are represented in Table 1.

In study [18], six data mining tools, namely, Orange, Weka, RapidMiner, KNIME, MATLAB, and ScikitLearn, were compared using six machine learning techniques: logistic regression, SVM, KNN, ANN, naïve Bayes, and random forest. Heart disease was classified using a dataset with 13 features, one target variable, and 303 instances (139 with cardiovascular disease and 164 healthy).

The dataset related to heart diseases in [26] comprises 70,000 patient records with 12 features, including age, height, weight, gender, systolic and diastolic blood pressure, cholesterol, glucose, smoking status, alcohol consumption, physical activity, and the presence of cardiovascular disease (“cardio”: 1 for disease; 0 for healthy).

The dataset [13,19] of this study selected and used 14 specific features. In these studies, different preprocessing methods were implemented, using 14 characteristics but combining them into 4 groups with around 6 features, each with different machine learning models.

In the case of [27,28], a dataset pertaining to cardiovascular diseases from Kaggle is employed. This dataset comprises twelve attributes, with a clear target variable. These attributes span across age, height, weight, gender, blood pressures, cholesterol and glucose levels, smoking and drinking habits, physical activity, and the presence or absence of cardiovascular disease.

The machine learning algorithms employed in these articles encompass decision tree (DT), naïve Bayes (NB), random forest (RF), k-Nearest neighbor (KNN), SVM (Linear Kernel), and logistic regression (LR). Additionally, boosted trees (GBT) are utilized as part of the analysis.

## 3. Materials and Methods

The heart disease dataset utilized in this research, sourced from the Kaggle platform (San Francisco, CA, USA) and named “Heart Failure”, serves as a consolidated resource for studying cardiovascular conditions. This dataset consists of approximately 10 crucial features for the classification of cardiovascular diseases. The dataset analysis and model development were conducted in a collaborative environment using Google Colab (Mountain View, CA, USA), leveraging its computational resources for efficient data handling and experimentation. Python 3.13 (Python Software Foundation, Wilmington, DE, USA) was employed as the primary programming language due to its versatility and the wide range of libraries available for data analysis and machine learning.

### 3.1. Data Analysis and Preprocessing

The dataset used combined five independent datasets on heart disease, resulting in a database with a total of 918 records and 11 features. These features include age, sex, chest pain type, resting blood pressure, cholesterol levels, fasting blood sugar, resting ECG, peak heart rate, exercise-induced angina, ST depression, and ST slope. This dataset represents the largest publicly accessible dataset on heart disease and provides a solid basis for research and analysis. This dataset can be seen in more detail in Table 2.

**Figure 1:**
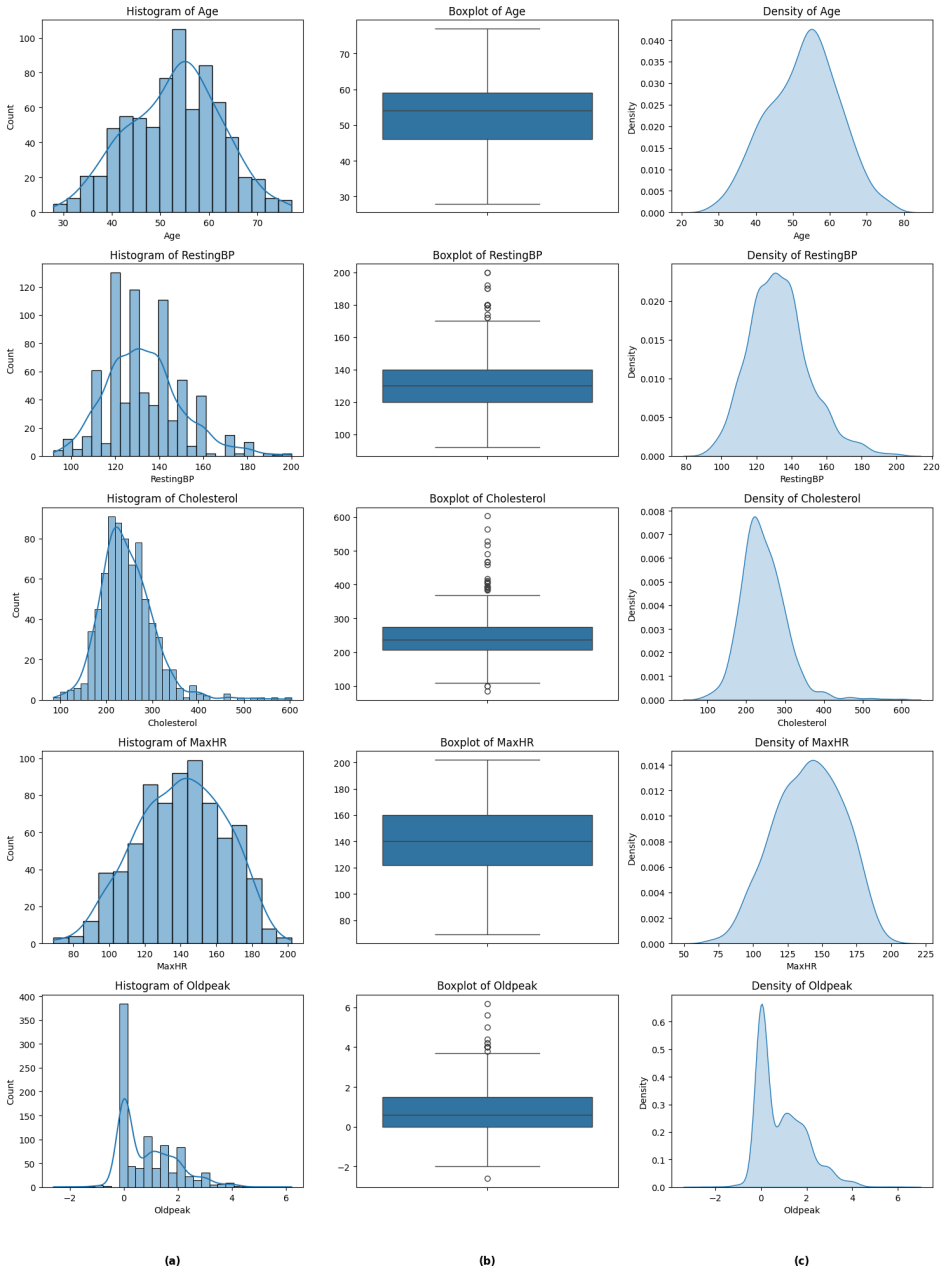
Distribution of key variables in a population sample. (**a**) Histograms illustrating the distribution of age (40–60 years), resting blood pressure (120–140 mmHg), cholesterol levels (200–300 mg/dL), maximum blood pressure (140–160 mmHg), and oldpeak (0–0.1 mV). (**b**) Boxplots displaying the variability and central tendencies of the same variables. (**c**) Density plots highlighting the shape of the distribution for each variable.

The preprocessing stage included a detailed analysis of the dataset. Exploratory data analysis (EDA) techniques, such as histograms, box plots, and density plots (Figure 1), were first employed to visualize the distribution of variables and detect anomalies. In data cleaning, 16 outliers were identified and eliminated using the interquartile range (IQR) method, a statistical approach chosen for its simplicity and effectiveness in detecting extreme deviations [29]. Clinical validation corroborated that these outliers corresponded to data errors or outlier conditions. Removing them allowed machine learning models to be trained on representative data, improving their reliability and clinical relevance.

After the database was cleaned, categorical variables were analyzed to identify those with low frequencies. Although a threshold of 5% was defined to identify infrequent categories, none of the categories met this criterion in the columns analyzed; therefore, all the original categories were retained. Ordinal coding was applied to categorical variables with a natural order, and one-hot coding was used to transform the remaining variables into numerical representations suitable for machine learning models.

**Figure 2:**
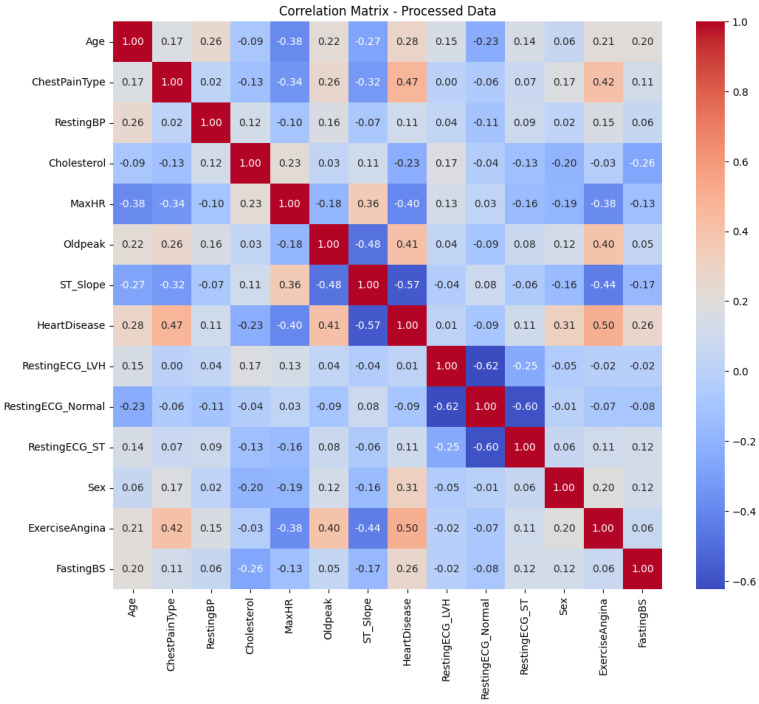
Correlation matrix.

The binary variables generated during this process were consolidated to simplify the dataset and standardize the representations. Finally, all columns with Boolean values were converted into binary representations (0 and 1), ensuring uniformity in the dataset format. Once these transformations were completed, a correlation matrix (Figure 2) was calculated to analyze the relationships between features in the processed dataset. This analysis made it possible to identify clearer patterns and more consistent relationships among the variables, validating the effectiveness of the preprocessing process, as multicollinearity issues were not present, particularly for models like logistic regression.

Each cell in the matrix represents the correlation between two variables, measured on a scale from −1 to 1. A value of 1 indicates a perfect positive correlation, −1 a perfect negative correlation, and 0 signifies no correlation.

In this matrix, a positive correlation is observed between age and heart disease, implying that the risk of heart disease increases with age. Similarly, a negative correlation is noted between cholesterol levels and heart disease, indicating that lower cholesterol levels are associated with a reduced risk of heart disease. Other variables, such as resting blood pressure, fasting blood sugar, and maximum heart rate achieved, also show positive correlations with heart disease. On the other hand, exercise-induced angina and depression of the ST segment are negatively correlated with heart disease.

Overall, this correlation matrix shows that variables associated with health risks, such as older age, high blood pressure, high cholesterol, and diabetes, are also linked to a higher risk of heart disease.

### 3.2. Analyzing Our Target Feature

**Figure 3:**
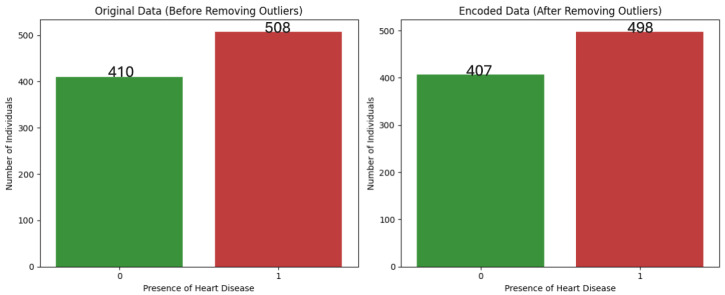
Comparison of target feature distribution before and after removing outliers.

The target variable in this study represents the presence or absence of heart disease, where 1 indicates the presence of the condition and 0 represents its absence. Initially, the dataset contained 508 instances of heart disease and 410 cases without it, as shown in Figure 3. After removing outliers, the distribution slightly changed to 498 instances of heart disease and 407 cases without it. Despite this slight imbalance, the dataset remained reasonably balanced overall.

**Figure 4:**
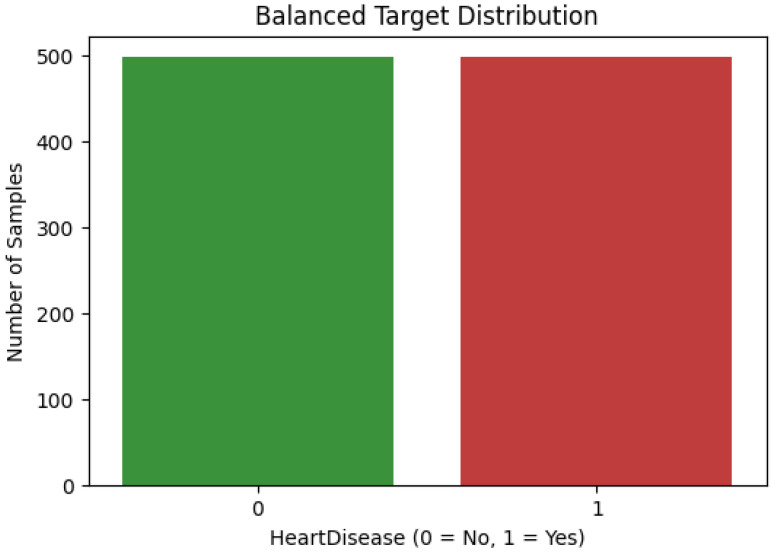
Balanced distribution of the target feature after applying oversampling techniques.

Before classification with the machine learning models, the dataset was balanced using oversampling techniques. The minority class (absence of heart disease) was resampled to match the size of the high class. The result was a balanced dataset with 498 samples for each class, as shown in Figure 4. By equalizing the number of samples from both classes, the model training process can effectively reduce potential biases and improve the accuracy of predictions for both conditions.

The final balanced distribution of the target variable ensures sufficient data representation for both classes, enabling the classification model to be trained effectively. This preprocessing step strengthens the reliability of the model’s evaluation and increases its capacity to generalize well to unseen data.

### 3.3. Machine-Learning Methods Used in This Study

In this section, a concise description of the machine-learning classification algorithms employed in the research is presented. These algorithms include LR, DT, RF, k-NN, and MLP.

#### 3.3.1. Logistic Regression (LR)

**Figure 5:**
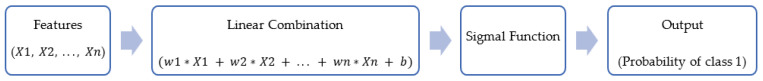
Architecture of the logistic regression (LR) model.

LR is a multivariable method widely used to model the relationship between multiple independent variables and a categorical dependent variable (Figure 5). It is the statistical method of choice when predicting the occurrence of a binary outcome (see Figure 1), such as determining whether someone is sick or healthy or making yes-or-no decisions [30]. This approach is commonly applied in situations involving disease states and decision-making. In statistical terms, LR is used to solve binary classification problems by modeling events and classes probabilistically through logistic functions [31,32].

#### 3.3.2. Decision Tree (DT)

**Figure 6:**
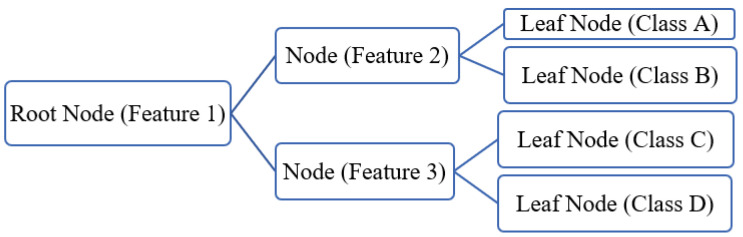
Architecture of the decision tree (DT) model.

DTs are sequential models that logically combine a series of simple tests; each test compares a numeric attribute against a threshold value or a nominal attribute against a specific set of values [33]. DTs serve as versatile prediction and classification mechanisms, recursively dividing a dataset into subsets based on the values of associated input fields or predictors [34]. This subdivision creates partitions and descendant nodes, known as leaves, containing internally similar target values, and as one descends the tree, increasingly different values between leaves (Figure 6). This fundamental characteristic of DTs allows them to adapt to diverse situations, making them crucial tools in the fields of decision-making and predictive analysis [35].

#### 3.3.3. Random Forest (RF)

**Figure 7:**
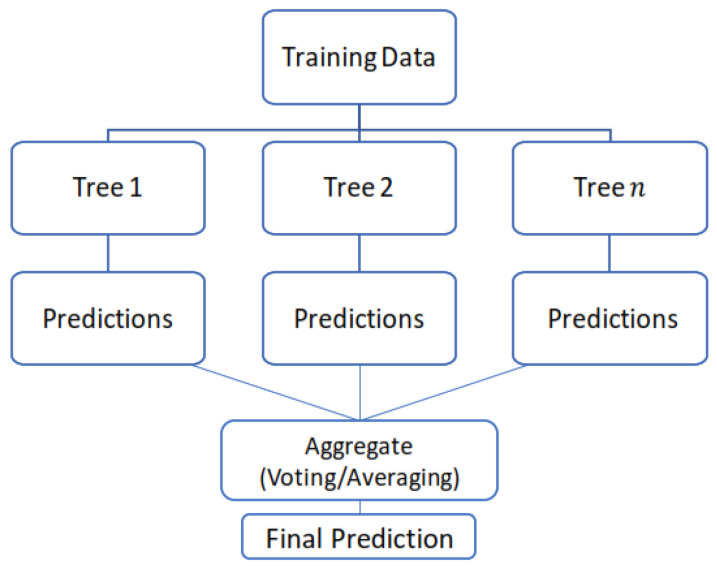
Architecture of the random forest (RF) model.

RF is an ensemble machine-learning technique that combines multiple individual DTs to create a more robust and accurate predictive model [36]. It operates by constructing a multitude of DTs during training and outputs the mode of the classes (classification) or the mean prediction (regression) of the individual trees as can be seen in its architecture in Figure 7. The randomness introduced in the tree-building process, both in terms of the data samples used for training and the features considered at each split, enhances the model’s generalization ability and reduces overfitting, yielding improved overall performance and reliability in predicting outcomes for new data points [37,38].

#### 3.3.4. K-Nearest Neighbor (KNN)

**Figure 8:**
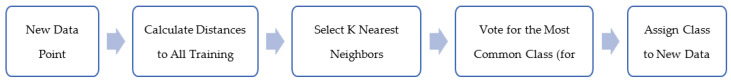
Architecture of the k-nearest neighbors (k-NN) model.

KNN is a classification and regression algorithm used in machine learning. In classification, k-NN assigns a class to a data point based on the classes of its nearest neighbors in a feature space [39]. In regression, k-NN estimates the numerical value of a data point by taking the average of its nearest neighbors’ values (Figure 8). The “k” in k-NN represents the number of neighbors considered when making the prediction, and the choice of this value affects the model’s accuracy [40]. K-NN is a simple and effective method, especially for data in which decision boundaries are nonlinear or when the underlying data structure is complex and cannot be easily modeled using mathematical equations [41].

#### 3.3.5. The Multi-Layer Perceptron (MLP)

**Figure 9:**
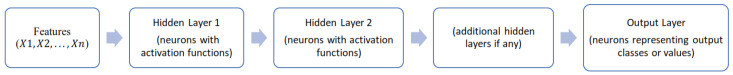
Architecture of the multi-layer perceptron (MLP) model.

MLP is a kind of artificial neural network that has been designed for supervised learning tasks. This consists of multiple layers of nodes, which include an input layer through which the network receives the initial data, one or more hidden layers that process this information, as well as an output layer that produces the final prediction or classification [42]. Each node in the network is a perceptron, applying a mathematical transformation to its inputs and passing the result to the next layer (see Figure 9). MLPs are able to learn complex patterns and relations in data, which makes them suitable for tasks such as regression, classification, and pattern recognition. Techniques such as backpropagation are used to adjust the weights of connections between nodes during training, allowing the network to learn and improve its performance over time [43,44,45].

## 4. Experimental Results

In this section, five machine-learning techniques were used to carry out an efficient extraction of features and classify heart failure diagnoses. These techniques include LR, RF, DT, k-NN, and the MLP classifier. The dataset was divided into two segments: 80% for the training phase and 20% for the testing process. To consider these models with high specificity requires it to be (≥0.90) to minimize false positives, an excellent AUC (≥0.90) indicating near perfect discrimination between classes [46]. A low Brier score (≥0.05) reflecting very accurate predictions, and a Matthews correlation coefficient (MCC) approaching +1 indicates excellent performance, close to 0 suggests that the model is random, and negative values indicate a complete misclassification. Values lower than these are indicative of a poorly performing model [47,48].

### 4.1. Model Building and Performance Evaluation

#### 4.1.1. Logistic Regression (LR)

The logistic regression results show in Table 3, with an F1 Score of 0.85 for both classes, the model maintains an adequate balance between precision and sensitivity. Furthermore, the overall accuracy of 85% indicates that the model correctly classifies most of the samples in a set with similar support between classes (102 and 98 for classes 0 and 1, respectively). The similar values of precision and recall for both classes indicate that the model does not exhibit a significant preference toward any specific class.

An AUC of 0.94 is obtained, demonstrating excellent class discrimination capability. The Brier score of 0.10 and MCC of 0.70 indicate good calibration of the prediction probabilities and a positive correlation between the real and predicted labels.

#### 4.1.2. Decision Tree (DT)

The decision tree results shown in Table 4 display an F1 score of 0.90 for class 0 and 0.89 for class 1, indicating a well-balanced model that effectively handles both classes. The overall accuracy of 90% highlights the model’s ability to correctly classify a large majority of samples, with support fairly evenly distributed across classes (102 for class 0 and 98 for class 1). The similar precision and recall values suggest that the model does not exhibit a significant preference towards one class over the other.

An AUC of 0.89 shows the model’s ability to discriminate between classes, while the Brier score of 0.10 and MCC of 0.79 indicate good calibration of predicted probabilities and a strong positive correlation between actual and predicted labels. Furthermore, the specificity of 0.94 highlights the model’s effectiveness in identifying true negatives.

#### 4.1.3. Random Forest (RF)

The outcomes of the random forest technique indicate strong performance in Table 5, with the precision, recall, and F1 score evenly distributed across both classes: class 0 (90% precision, 93% recall, 92% F1) and class 1 (93%, 90%, 91%). The total accuracy of 92% along with the macro and weighted averages indicate that the model performs effectively in balanced distributions.

The 93% specificity suggests that the model accurately recognizes the majority of negative cases, while an AUC of 0.97 demonstrates its ability to differentiate between categories effectively. Additionally, the low Brier score (0.06) indicates that the predicted probabilities are accurately calibrated, whereas the MCC of 0.83 demonstrates that the model is dependable, despite a minor imbalance in the classes.

#### 4.1.4. K-Nearest Neighbor (KNN)

In this part of the KNN method, they show an acceptable performance in Table 6. First, in class 0, it has an accuracy of 78%, a recall of 69%, and an F1 score of 73%. On the other hand, in class 1, the accuracy decreases (71%), but the recall increases (80%), with an F1 score of 75%. Therefore, it is difficult for the model to correctly identify the classes, resulting in an overall accuracy of 74%.

A specificity of 69% indicates that the model has trouble correctly identifying negative cases, while an AUC of 0.81 reflects a reasonable ability to differentiate between classes. However, the Brier score (0.18) indicates that the predicted probabilities are not perfectly calibrated. The Matthews correlation coefficient (MCC) of 0.48 suggests moderate performance, especially in scenarios with a slight imbalance between classes.

#### 4.1.5. Multi-Layer Perceptron (MLP)

The MLP model shows solid performance in Table 7, with an accuracy of 94% and an F1 score of 84% for class 0, although the lower recall (75%) indicates difficulties in identifying some positive cases. In contrast, for class 1, the recall is high (95%), but the accuracy decreases to 79%, resulting in an F1 score of 86%. These differences reflect a balance between the identification of both classes, with an overall accuracy and average metrics of 85%.

Additionally, the model presents a moderate specificity (75%), suggesting some difficulty in correctly recognizing negative instances. However, the AUC of 0.95 and the Brier score of 0.11 highlight the model’s ability to distinguish between classes and generate well-calibrated probabilities. The MCC of 0.72 indicates solid performance.

### 4.2. Summary Model Building and Performance Evaluation

Table 8 provides a summary of the accuracy, recall, and F1 scores of the employed methods. This table gives a summary view of the performance of each method in relation to precision and other critical metrics, allowing for a comparative evaluation of their performance in the study.

### 4.3. Statistical Significance Analysis

**Figure 10:**
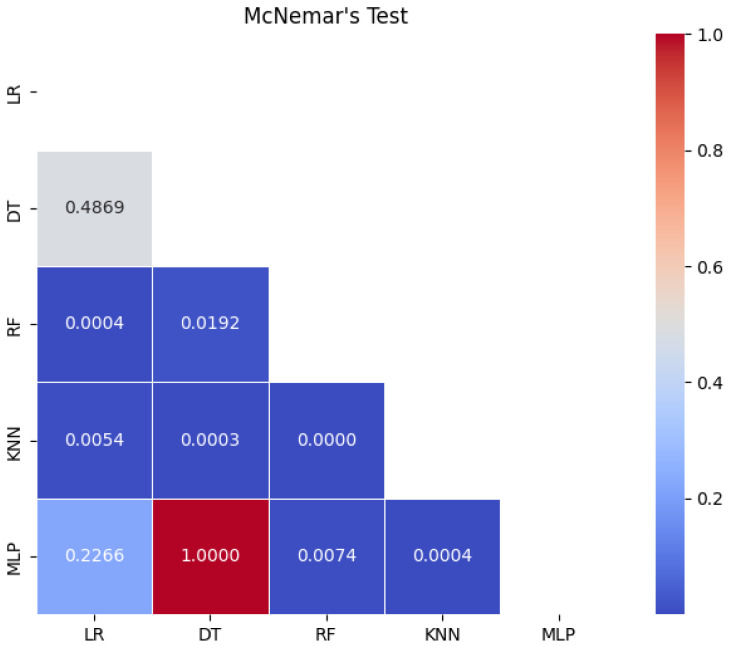
Statistical significance matrix from McNemar’s test comparing classifier pairs. Each cell represents the *p*-value for the comparison between two models, with darker shades indicating lower *p*-values (greater statistical significance).

To statistically validate the differences in performance between classifiers, McNemar’s test was applied to the paired predictions of all models. The resulting significance matrix (Figure 10) reveals strong contrasts: random forest showed statistically superior performance compared to logistic regression (p=0.0005) and K-nearest neighbors (p<0.0001), in line with their main metrics (AUC = 0.97; specificity = 0.93). In contrast, KNN performance was systematically inferior, showing significant differences against all models (p<0.01). Notably, no significant differences were observed between logistic regression and decision tree (p=0.5716) or MLP (p=0.1796), suggesting overlapping efficacy in specific classification scenarios.

## 5. Discussion

In the present research, the Heart Failure Prediction dataset was used to test the optimal machine learning classification algorithm for efficient feature removal and diagnosis of heart failure. To this end, different machine learning algorithms were analyzed to identify their strengths and weaknesses. Logistic regression (LR) shows a balanced performance, with an F1 of 0.85 and an AUC of 0.94, indicating its ability to differentiate between classes. However, although it is robust in both classes, its metrics are not as competing as those of other models evaluated.

Decision tree (DT) outperforms LR in accuracy (90%) and achieves a better balance between precision and recall. Its F1 for class 0 is solid, reaching 0.90, and its high specificity (0.94) highlights its effectiveness in identifying true negatives. However, its AUC of 0.89 is slightly lower than that of LR and other models, suggesting that it can still improve in discriminating between classes.

The k-nearest neighbors (k-NN) model faces overall difficulties, with a lower F1 (0.75) and reduced specificity (0.69), reflecting problems in handling class imbalances and recognizing negative cases. Although its AUC of 0.81 is acceptable, it falls short of the other models evaluated.

The multilayer perceptron (MLP) offers competitive performance, standing out with an AUC of 0.95 and an overall accuracy of 85%. However, the disparity in recall (75% for class 0 and 95% for class 1) evidences difficulties in consistently identifying positive instances in both classes, resulting in a lower F1 compared to the RF model.

Finally, the random forest (RF) model performs remarkably well, achieving a precision of 90% for class 0 and 93% for class 1, demonstrating its ability to accurately identify both positive and negative cases. Additionally, recall is also high, with 93% for class 0 and 90% for class 1, ensuring that most correct instances are detected. This results in F1 scores of 0.92 and 0.91 for classes 0 and 1, respectively, reflecting a well-balanced overall performance.

Moreover, metrics such as specificity (0.93) and AUC (0.97) highlight the model’s ability to effectively differentiate between classes while minimizing errors. The Brier score of 0.06 confirms that the predicted probabilities are highly reliable, and the MCC of 0.83 indicates strong and balanced performance, even in scenarios with potential class imbalances. These results position random forest as the best choice among the evaluated models. This model was found to be able to reliably identify cases of heart failure.

In the comparison of studies on machine learning models (Table 9), our approach stands out by achieving the highest precision (92%) through correct preprocessing and hyperparameter tuning. This contrasts with other studies that did not handle outliers and obtained lower precisions, such as study [18] with 85% [13], with 83% [27], with 76%, and study [28] with 73%. Our result suggests that data preprocessing, specifically outlier management, can significantly improve model performance. Study [26], which also managed outliers, achieved a high precision of 89.58%, and study [19] achieved a precision of 91.70% supporting this observations.

A major finding of our study is that despite using only 12 features for the model, our accuracy was higher (92%) compared to other studies that used 14 features. For example, study [19] achieved an accuracy of 91.70% with 14 features, while study [13] recorded 83% using the same amount of features. This shows that adequate feature selection and effective preprocessing, such as outlier treatment, can enhance model performance significantly even with fewer features.

The McNemar test significance matrix provides a rigorous statistical basis for comparing the effectiveness of the models. The dominance of random forest (*p* < 0.001 versus key models) underscores its ensemble advantage, which mitigates overfitting by aggregating diverse decision trees, thus capturing complex feature interactions more effectively. In contrast, the poor performance of KNN (*p* < 0.01 in all comparisons) highlights its limitations in clinical datasets, where noise and class imbalance degrade its distance-based logic. The lack of significant differences between logistic regression, decision tree, and MLP (*p* ≥ 0.05) may reflect their shared sensitivity to shallow linear or nonlinear decision boundaries in this dataset.

The algorithm is able to integrate with devices that provide static data or electronic medical records (EHRs), digital systems that store patients’ medical history, laboratory results, and patient diagnoses. This assists healthcare professionals in making informed decisions and optimizing the treatment of cardiovascular or chronic patients. When linked to EHRs, the algorithm processes medical history and laboratory results to identify high-risk patients. In the ED, models with high sensitivity (MLP at 94%) prioritize critical cases, while accurate algorithms (DT at 93%) reduce false positives and avoid unnecessary testing. In additional, continuous monitoring would detect patterns of early deterioration, triggering alerts for timely interventions and optimizing the allocation of hospital resources.

This study demonstrates promising results; however, certain limitations must be recognized. First, although the dataset provides a solid basis for analysis, its extension with additional variables-such as left ventricular ejection fraction (LVEF) or biomarkers such as NT-proBNP-could improve clinical relevance and diagnostic granularity. Second, although the sample size is sufficient for robust model training, incorporating data from diverse populations and comorbid conditions would improve generalizability across demographics.

Importantly, the models were validated on a preprocessed static dataset and have not yet been tested in real-world clinical workflows. Factors such as variability in real-time data collection or evolving patient conditions could affect performance.

## 6. Conclusions

In summary, this study emphasizes the effectiveness of machine learning algorithms in the classification of heart failure, with the random forest model achieving the highest accuracy at 92% and standing out in distinguishing between positive and negative cases. The advanced preprocessing techniques, such as outlier removal and class balancing, significantly improved the performance of the model. Machine learning’s ability to handle complex data and provide accurate diagnoses is crucial for early medical intervention and improving patients’ quality of life.

In comparison to previous studies, this work excels in accuracy due to efficient data processing and hyperparameter optimization. It shows that a well-processed dataset, even with fewer features, can outperform studies where data processing was not prioritized. In conclusion, machine learning not only strengthens its role in medicine but also shows great potential to transform the diagnosis and treatment of cardiovascular diseases. A promising future application is the integration of these models with real-time monitoring devices. Future work should prioritize prospective validation in hospital settings to test the model’s adaptability in dynamic clinical workflows, particularly for reducing false positives and prioritizing critical cases. Additionally, exploring hybrid architectures (e.g., combining random forest with LSTM networks) could capture temporal patterns in continuous ECG data, enhancing accuracy in scenarios requiring both static and dynamic feature analysis.

## Figures and Tables

**Table 1:** Models used in classifying the presence or absence of cardiac conditions.

Description	P. Languages	Model	Precision	Recall	F1 Score	Cite
Six data mining tools and machine learning techniques were applied	Orange (3.34), Weka (3.8.3), Knime (4.7), Matlab (R2023a), Scikit-learn (1.2.x)	SVM	83.84%	78.83%	-	[18]
		KNN	76.43%	77.37%	-	
		NB	84.51%	81.02%	-	
		RF	84.48%	81.75%	-	
		ANN *	85.86%	83.94%	-	
		LR	83.84%	81.75%	-	
12 features, GridSearchCV, Hyperparameter tuning	Python (3.10)	XGB	88.93%	83.57%	86.16%	[26]
		MLP	88.70%	84.85%	86.71%	
		DT *	89.58%	81.61%	85.42%	
		RF *	89.42%	81.61%	86.32%	
The study classifies heart diseases using a dataset from the UCI database. Using 14 characteristics	Python (3.10)	DT	77.80%	75.50%	86.40%	[19]
		NB	85.40%	80.40%	86.50%	
		RF	88.70%	83.20%	87.90%	
		KNN *	91.70%	94.80%	90.80%	
		SVM	90.70%	87.80%	88.50%	
		LR	88.10%	86.10%	86.50%	
Evaluates multiple ML classifiers for diagnosing using 14 characteristics grouped into 4 categories	Python (3.8)	LR	-	75%	-	[13]
		KNN	-	64%	-	
		SVM	-	70%	-	
		SVML	-	75%	-	
		NB	-	78%	-	
		DT *	-	83%	-	
Classify cardiovascular data using 12 features to predict the presence or absence of the disease	-	NB	70%	34%	46%	[27]
		DT	75%	68%	71%	
		LR	73%	68%	70%	
		RF	71%	71%	71%	
		SVM *	76%	64%	69%	
		KNN	66%	64%	65%	
Classify cardiovascular data using 12 features to predict the presence or absence of cardiovascular disease	-	KNN	70%	70%	70%	[28]
		SVM *	73%	71%	71%	
		DT	63%	63%	63%	
		ANN *	73%	73%	73%	
		NB	71%	71%	71%	
		RF	70%	70%	70%	
		LR *	73%	73%	73%	

* Algorithm with the highest precision.

**Table 2:** Characteristics of the cohort used in classifying the presence or absence of cardiac conditions.

SN	Attribute Name	Type	Description	Mean ± Std Dev	Min–Max	Categories (Frequency)	Units
1	Age	Numerical	Age of the patient	53.51 ± 9.43	28.0–77.0	-	years
2	Sex	Categorical	Sex of the patient	-	-	M: 725 (79%), F: 193 (21%)	-
3	ChestPainType	Categorical	Type of chest pain experienced	-	-	ASY: 496 (54%), NAP: 203 (22%), ATA: 173 (19%), TA: 46 (5%)	-
4	RestingBP	Numerical	Resting blood pressure measured	132.40 ± 18.51	0.0–200.0	-	mm Hg
5	Cholesterol	Numerical	Serum cholesterol level	198.80 ± 109.38	0.0–603.0	-	mg/dL
6	FastingBS	Binary	Fasting blood sugar > 120 mg/dL	0.23 ± 0.42	0.0–1.0	0: 704 (77%), 1: 214 (23%)	-
7	RestingECG	Categorical	Resting electrocardiogram results	-	-	Normal: 552 (60%), LVH: 188 (21%), ST: 178 (19%)	-
8	MaxHR	Numerical	Maximum heart rate achieved	136.81 ± 25.46	60.0–202.0	-	bpm
9	ExerciseAngina	Binary	Presence of exercise-induced angina	-	-	N: 547 (60%), Y: 371 (40%)	-
10	Oldpeak	Numerical	ST depression induced by exercise relative to rest	0.89 ± 1.07	−2.6–6.2	-	-
11	ST_Slope	Categorical	The slope of the peak exercise ST segment	-	-	Flat: 460 (50%), Up: 395 (43%), Down: 63 (7%)	-
12	HeartDisease	Binary	Output class	0.55 ± 0.50	0.0–1.0	1: 508 (55%), 0: 410 (45%)	-

**Table 3:** Logistic regression (LR) method output based on precision, recall, and F1 score.

	Precision	Recall	F1 Score	Support
0	0.86	0.84	0.85	102
1	0.84	0.86	0.85	98
Accuracy			0.85	200
Macro Avg.	0.85	0.85	0.85	200
Weighted Avg.	0.85	0.85	0.85	200

Additional metrics: Specificity = 0.84; AUC = 0.94; Brier score = 0.10; MCC = 0.70.

**Table 4:** Decision tree (DT) method output based on precision, recall, and F1 score.

	Precision	Recall	F1 Score	Support
0	0.86	0.94	0.90	102
1	0.93	0.85	0.89	98
Accuracy			0.90	200
Macro Avg.	0.90	0.89	0.89	200
Weighted Avg.	0.90	0.90	0.89	200

Additional metrics: Specificity = 0.94; AUC = 0.89; Brier score = 0.10; MCC = 0.79.

**Table 5:** Random forest (RF) method output based on precision, recall, and F1 score.

	Precision	Recall	F1 Score	Support
0	0.90	0.93	0.92	102
1	0.93	0.90	0.91	98
Accuracy			0.92	200
Macro Avg.	0.92	0.91	0.91	200
Weighted Avg.	0.92	0.92	0.91	200

Additional metrics: Specificity = 0.93; AUC = 0.97; Brier score = 0.06; MCC = 0.83.

**Table 6:** K-nearest neighbors (KNN) method output based on precision, recall, and F1 score.

	Precision	Recall	F1 Score	Support
0	0.78	0.69	0.73	102
1	0.71	0.80	0.75	98
Accuracy			0.74	200
Macro Avg.	0.74	0.74	0.74	200
Weighted Avg.	0.74	0.74	0.74	200

Additional metrics: Specificity = 0.69; AUC = 0.81; Brier score = 0.18; MCC = 0.48.

**Table 7:** Multi-layer perceptron (MLP) method output based on precision, recall, and F1 score.

	Precision	Recall	F1 Score	Support
0	0.94	0.75	0.84	102
1	0.79	0.95	0.86	98
Accuracy			0.85	200
Macro Avg.	0.86	0.85	0.85	200
Weighted Avg.	0.87	0.85	0.85	200

Additional metrics: Specificity = 0.75; AUC = 0.95; Brier score = 0.11; MCC = 0.72.

**Table 8:** Overall summary of the methods used and their precision, recall, and F1 scores.

ML Algorithms		Confusion	Matrix	Precision	Recall	F1 Score
		0	1			
LR	0	86	16	0.84	0.86	0.85
	1	14	84			
DT	0	96	6	0.93	0.84	0.88
	1	16	82			
RF *	0	94	8	0.92	0.90	0.91
	1	10	88			
KNN	0	70	32	0.71	0.80	0.75
	1	20	78			
MLP	0	72	30	0.75	0.94	0.84
	1	6	92			

* The best model from our study.

**Table 9:** Comparison of the best model in the present study with the best model in previous re-search.

Study	Features	Outlier Handling	Best Precision
Our	12	Identified and Removed	92%
[18]	13	No	85.86%
[26]	12	Identified and Removed	89.58%
[19]	14	Identified and Removed	91.70%
[13]	14	No	83%
[27]	12	No	76%
[28]	12	No	73%

## Data Availability

https://www.kaggle.com/datasets/fedesoriano/heart-failure-prediction/data (accessed on 1 February 2025).
